# Quantifying the Antiviral Effect of IFN on HIV-1 Replication in Cell Culture

**DOI:** 10.1038/srep11761

**Published:** 2015-06-29

**Authors:** Hiroki Ikeda, Ana Godinho-Santos, Sylvie Rato, Bénédicte Vanwalscappel, François Clavel, Kazuyuki Aihara, Shingo Iwami, Fabrizio Mammano

**Affiliations:** 1 Department of Biology, Kyushu University, Fukuoka 812-8581, Japan; 2 INSERM, U941, Paris, France; 3 Univ Paris Diderot, Sorbonne Paris Cité, IUH, Paris, France; 4 Institute of Industrial Science, The University of Tokyo, Meguro-ku, Tokyo, Japan; 5 Graduate School of Information Science and Technology, The University of Tokyo, Bunkyo-ku, Tokyo, Japan; 6 PRESTO, JST, Kawaguchi, Saitama 3320012, Japan; 7 CREST, JST, Kawaguchi, Saitama 3320012, Japan

## Abstract

Type-I interferons (IFNs) induce the expression of hundreds of cellular genes, some of which have direct antiviral activities. Although IFNs restrict different steps of HIV replication cycle, their dominant antiviral effect remains unclear. We first quantified the inhibition of HIV replication by IFN in tissue culture, using viruses with different tropism and growth kinetics. By combining experimental and mathematical analyses, we determined quantitative estimates for key parameters of HIV replication and inhibition, and demonstrate that IFN mainly inhibits *de novo* infection (33% and 47% for a X4- and a R5-strain, respectively), rather than virus production (15% and 6% for the X4 and R5 strains, respectively). This finding is in agreement with patient-derived data analyses.

Viral infections trigger innate immune responses in the host, including the production of type-I interferons (IFNs) by several cell types, including specialized immune cells. IFNs, by binding receptors on a target cell surface, induce the expression of hundreds of interferon-stimulated genes (ISGs). Some ISGs have direct antiviral activity against a range of pathogens including the human immunodeficiency virus type-1 (HIV-1), while others link innate immunity to adaptive immunity (reviewed in^1^,^2^). Various studies have collectively demonstrated that IFNs constrain multiple steps of HIV-1 replication cycle, from virus entry to the release of newly synthesized virions (e.g.^3^,^4^,^5^,^6^,^7^,^8^,^9^,^10^,^11^,^12^), resulting in potent inhibition of virus replication in cell culture.

In HIV-infected patients, IFN levels in serum follow a biphasic curve, with elevations observed both in early infection and in advanced disease (reviewed in^13^). Early clinical trials evaluating the effect of exogenously administered IFN on HIV-1 infection showed clinical benefit in some, but not all studies^14^,^15^,^16^. IFN administration to patients not receiving antiretroviral therapy generally results in significant, but clinically unsatisfactory, reductions in HIV-1 viremia^17^,^18^, and was shown to delay viral rebound following antiretroviral treatment interruption^19^,^20^.

Previous studies have shown the advantage of coupling mathematical modeling to *in vitro* experimental data, leading to robust quantification of virus infection parameters^21^,^22^,^23^. Here we took advantage of this strategy to explore two aspects of the inhibition of HIV replication by IFN. First, we quantified the potency of IFN inhibition on HIV replication in culture, by comparing viruses with different tropism and growth kinetics. Second, since IFN is known to act both on early and late steps of HIV replication, we used mathematical modeling to explore whether the dominant effect of IFN is to prevent new infections or to thwart virus production. Our analyses provide quantitative estimates for several parameters of HIV replication and inhibition, and shows that IFN primarily inhibits *de novo* infection.

## Results and Discussion

### Characterizing target cell dynamics

**Figure 1:**
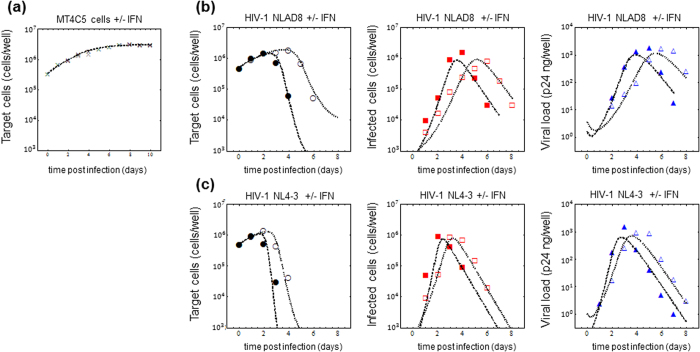
Dynamics of MT4C5 cells and HIV-1 replication in the absence and presence of IFN. Three hundred thousand MT4C5 cells were seeded in 24-well plate in two milliliter of a culture medium with and without IFN in **(a)**. Cells were counted daily for 10 days, the growth kinetics of MT4C5 cells in these conditions were estimated as described in text. Black and gray crosses represent the typical cell growth in the absence and presence of IFN, respectively. The numerically estimated parameters are *g* = 0.68 ± 0.03 per day and *T*_*max*_ = 3.01 × 10^6^ ± 0.09 × 10^6^ cells per well regardless of IFN. Furthermore, MT4C5 cells were inoculated with 10 ng of p24 of NL-AD8 in **(b)** and HIV-1 NL4-3 in **(c)** without or with IFN. The number of intracellular Gag-negative and -positive MT4C5 cells per well and the amount of p24 viral protein (ng/well) in the culture supernatant were measured daily from *t* = 0 to 8. The bullet (●■▲) and open (○□△) symbols show the representative experimental data from one experiment in the absence and presence of IFN, respectively. The best fit of the mathematical model, Eqs. (2, 3, 4), to the data is depicted as broken and dotted lines in the absence and presence of IFN, respectively.

To quantitatively predict the antiviral effect of IFN on HIV-1 replication, we first estimated the growth kinetics of MT4C5 cells, which have been commonly used for HIV-1 studies^24^,^25^,^26^, in the absence and presence of IFN-alpha 2b. MT4C5 cells are among the few T-cell lines that respond to IFN by inducing anti-HIV activities^27^. In addition, they allow the comparison of virus replication and inhibition for viruses characterized by different tropism, because they express the two major co-receptors used by HIV, the chemokine receptors CCR5 and CXCR4. *In vitro* cell growth kinetics were measured in the absence or presence of IFN at 50 IU/ml. This IFN concentration induces measurable inhibition of HIV replication without suppressing virus spread, allowing the quantification of virus growth kinetics in comparable conditions. Also, this concentration of IFN is in the range of those observed in the plasma of patients during acute HIV infection^28^. Cells were allowed to expand for 10 days, with daily replacement of medium (+/− IFN), and the total number of live cells was assessed. To estimate cell growth kinetics in cell culture, we used the following mathematical model^23^,^29^:


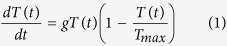


where the variable *T*(*t*) is the number of MT4C5 cells at time *t* and the parameter *g* and *T*_*max*_ are the growth rate of MT4C5 cells and the carrying capacity (maximum number of cells) of a 24-well plate, respectively. Cell growth analysis similar to those performed in^23^ revealed that for MT4C5 cells *g* = 0.63 ± 0.00 (mean ± standard deviation) and 0.58 ± 0.04 day^−1^ (*p* > 0.15, obtained by Welch’s t test), and *T*_*max*_ *=* (3.33 ± 0.16) × 10^6^ and (3.17 ± 0.29 × 10^6^ cells/well (*p* > 0.40) in the absence and presence of IFN, respectively (Fig. 1(a)). Thus, at the concentration used here, IFN did not affect cell proliferation. Note that we set *g *= 0 in our simulation if the number of MT4C5 cells decreases below *T*_*min*_ *=* 2.0 × 10^4^ cells/well. This is because the proliferation rate of MT4C5 cells is markedly diminished if the initial number of the cells is lower than *T*_min_ (i.e., *T*(0) ≤ *T*_*min*_) (data not shown).

### HIV-1 replication in cell culture in the absence and presence of IFN

We carried out MT4C5 infection experiments in the culture conditions described above. Infections were performed using the equivalent of 10 ng of p24 of two HIV reference strains, which differ for their tropism: NL4-3 (a CXCR4-using virus) and NL-AD8 (a CCR5-using virus). Virus spread in culture was monitored daily by evaluating the percentage of infected and uninfected cells (by intracellular Gag staining and FACS analysis), and viral production by p24 ELISA performed on cell supernatants. Each day the medium was replaced by fresh medium complemented or not by IFN.

As shown in Fig. 1b,c, the number of uninfected cells per well (left panels) initially increased, but then rapidly decreased, due to virus spread and to the cytopathic effect of virus infection. In the presence of IFN, cell proliferation persisted for 1–2 additional days, suggesting a partial protective effect of IFN. By comparing the kinetics of infected cells per well (middle panels) NL4-3 displayed faster propagation and resulted in approximately 10^6^ infected cells at day 2, while this number was reached one day later in cultures infected by NL-AD8. The number of infected cells then decreased, as a consequence of cell death. The partial inhibition exerted by IFN on both viruses delayed the peak of infected cells per well by 1 or 2 days, for NL4-3 and NL-AD8, respectively. Virus production in the culture supernatant (right panels) was consistent with the kinetics of infected cells, showing similar levels of p24 production for the two viruses, and again the IFN-induced delay. Interestingly, similar amounts of virus particles (as measured by p24/well) were produced in IFN-treated and untreated cultures, but in IFN-treated cultures virus production was delayed. It should be noticed that these levels of p24 correspond to the virus production accomplished during 24 h, because the complete culture medium was replaced every day.

### Modeling antiviral effect of IFN on HIV-1 replication

In order to analyze these time-course experimental data, we used a modified model of viral infection^22^,^29^,^30^,^31^,^32^, which includes the minimum threshold of the target cell proliferation, described by the following differential equations:


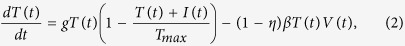



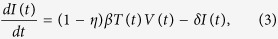



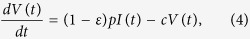


where *T*(*t*) and *I*(*t*) are the number of uninfected and infected cells per well of culture, respectively, and *V*(*t*) is the viral load measured by the amount of HIV-1 p24 per well, in the culture supernatant. The parameters *β*, *δ*, *p* and *c* represent the infection rate, the death rate of infected cells, the virus production rate, and the clearance rate of virions, respectively. The potential effects of IFN in this model are to reduce the *de novo* rate of infection by a fraction (1–*η*) and/or the production of virions from infected cells by a fraction (1–*ε*). In the absence of IFN, *η* = *ε* = 0; otherwise, *η* > 0 and *ε* > 0.

### Analyzing HIV-1 replication in cell culture

To analyze the replication of NL4-3 (or NL-AD8) strain, we simultaneously fit Eqs. (2, 3, 4) to all of the data from experimental conditions observed during time-course analyses: the number of intracellular Gag-positive and -negative cells in cultures treated or not with IFN, and the amount of p24 in the supernatants of these cultures, using nonlinear least-squares regression (using the FindMinimum package of *Mathematica9.0* that minimizes the sum of squared residuals (SSR, i.e., Eq. (5)): see **Methods**). Here we note that *g* and *T*_*max*_ were estimated to be 0.63 per day and 3.3 × 10^6^ cells per well from the cell growth experiments, respectively (see above). In addition, we used a clearance *c* value of 2.3 per day, which is estimated from daily harvesting of virus (i.e., the amount of p24 was reduced by approximately 90% per day by the daily medium-replacement procedure, and the degradation of p24 is negligible in our experimental time-scale). Also, the initial values for *T* and *I* at t = 0 (in the presence and absence of IFN) were fixed: *T*(0) = 5.8 × 10^5^ and *I*(0) = 0 cells/well. The remaining five parameters *β*,*δ*,*p*,*η* and *ε*, along with two initial values for *V*(0)s in the absence and presence of IFN for NL4-3 and NL-AD8, were determined by fitting the model to our experimental measurements. The estimated parameters of the model and derived quantities are given in Table 1, and discussed here below. The typical behavior of the model using these best-fit parameter estimates is shown together with the data from one experiment in Fig. 1b,c, which reveals that Eqs. (2, 3, 4) describe these *in vitro* data very well. Two additional experiments are shown in Supplemental Figure 1. These results suggest that the parameters that were estimated are representative of the various processes underlying the viral kinetics, including antiviral efficacy of IFN on HIV-1 infection.

As summarized in Table 1, our parameter estimation obtained from three independent experiments shows that HIV-1 NL4-3 strain replicates in target cells more efficiently than NL-AD8 strain. For example, the estimated value of the basic reproduction number *R*_0_ = (1–*η*)(1–*ε*)*βpT*(0)/*δ*c, which indicates the potential of virus infection (the number of cells infected by a typical infected cell)^31^,^32^, is greater for HIV-1 NL4-3 than for NL-AD8 strain. In addition, the Malthusian parameter 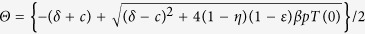, which indicates a fitness (or speed) of virus infection^31^,^32^, is also greater for HIV-1 NL4-3 than for NL-AD8 strain. These large differences are associated with co-receptor use of the NL4-3 strain and NL-AD8 strain^33^,^34^, and are consistent with tropism-associated viral pathogenesis *in vivo*^21^,^35^. Thus, we quantitatively interpreted the time-course data and extracted virus strain-specific kinetics.

### Antiviral effect of IFN on HIV-1 replication

Furthermore, we estimated the antiviral effects of IFN on *de novo* infection *η* *=* 0.33 ± 0.10 and 0.47 ± 0.06 for the NL4-3 strain and NL-AD8 strain, respectively. These values show relatively potent effect of IFN on this process. In contrast, the estimated antiviral effects of IFN on virus production are negligible: *ε* *=* 0.15 ± 0.11 and 0.06 ± 0.09 for the NL4-3 strain and NL-AD8 strain, respectively (see Table 1). In agreement with these findings, IFN efficiently reduces the basic reproduction number and the Malthusian parameter for both strains, but does not change the viral burst size (1–*ε*)*p*/*δ* (see Table 1). Taken together, our quantitative analyses strongly suggest that the major effect of IFN is to block *de novo* infection, rather than virus production or release, regardless of viral tropism. It is possible, however, that using higher IFN concentrations a more significant effect of IFN on virus production could be disclosed.

Supporting our conclusion, a previous report on the inhibition of HIV in IFN-treated patients showed better fit of their mathematical model under the assumption that the major effect of IFN is to prevent viral infection, rather than to inhibit virion production^36^. Interestingly, the major effect of IFN on HIV-1 infection is opposite to that on HCV infection, which has been also predicted by quantitative analyses of clinical data with mathematical models^30^,^37^.

### Conclusions

Using molecular and cell biology techniques, we are currently able to investigate the antiviral effects of IFN on different steps of the HIV-1 replication cycle. However, by these experimental approaches it remains difficult to interconnect those particular results in terms of the overall parameters defining a virus infection, like the replication rate or the burst size. By modeling *in vitro* time courses, and comparing the estimated parameters in the absence or presence of IFN, we could quantify the dominant role of IFN on several parameters. Our analysis showed that the major effect of IFN is to prevent viral infection, rather than to inhibit virion production in the cell cultures. These findings are consistent with the observation that IFN delayed the timing of the peak viral load but hardly changed the height of the peak (see Fig. 1b,c). Accordingly, *in silico,* the virus production rate, *p*, in Eqs. (2, 3, 4) affects the height of the peak viral load of *V*(*t*), while the infection rate, *β*, affects its timing.

One future challenge would be to quantitatively understand intracellular dynamics of HIV-1 replication, especially at the virus entry and post-entry steps using a mathematical model^38^,^39^. Complementary experimental and modeling approaches are required to determine the exact mechanism of action responsible for the antiviral effect of IFN on HIV-1 infection. Similar approaches can be envisaged to elucidate the antiviral effect of drugs or of the immune responses on a variety of viruses.

## Methods

### Cell culture, viruses and infection

MT4C5 cells^24^ were grown in RPMI-1640 medium supplemented with 10% heat-inactivated foetal bovine serum (FBS), 100 IU/mL of penicillin G, 100 μg/mL of streptomycin and 0.25 μg/ml of amphotericin B. HEK293T cells were cultured in DMEM medium supplemented with 10% FBS and antibiotics (100 IU/ml of penicillin G and 100 μg/ml of streptomycin). All cultures were maintained at 37°C in a humidified atmosphere with 5% CO_2_.

Growth kinetics of MT4C5 was measured in the absence and presence of IFN (IFN-alpha 2b, Immunotools, Friesoythe, Germany). 3 × 10^5^ cells were seeded in 2 ml of medium with and without IFN at 50 IU/ml (192 pg/ml) in 24-well plates. Cells were allowed to expand for 10 days, each day the culture medium was replaced by fresh medium with or without IFN, and the number of live cells was assessed through trypan-blue exclusion test. Viral production was performed by transfecting HEK293T cells with pNL4-3 or pNL-AD8 proviral constructs, using jetPEI (Polyplus transfection, Illkirch, France), following the manufacturers’ instructions. After 48 h, viral supernatant was collected and p24 production was measured using an HIV-1 p24 enzyme-linked immunosorbent assay (ELISA) kit (Innogenetics, Ghent, Belgium).

Infection experiments were conducted by plating MT4C5 cells as described above, and 24h later cells were exposed to virus supernatant containing 10 ng of p24. Cells were washed 2h post infection. Each day the total culture medium was replaced by fresh medium complemented or not by IFN. Time-course data were obtained for the following measurement: cell viability by trypan-blue exclusion; the number of infected and uninfected cells as measured by intracellular Gag staining and FACS analysis after gating the live cell population (Supplemental Figure 2), as in^25^; and virus particle production in the culture supernatant by p24 ELISA, as in^25^.

### Identification of best-fit parameters from experimental data

The total virus concentration in the supernatant and the number of uninfected and infected cells in the culture were measured every day for 8 days after infection of MT4C5 cells by NL4-3 or NL-AD8 strains of HIV-1, in the absence and presence of IFN. To estimate parameters in Eqs. (2, 3, 4) from three independent experiments, a nonlinear least-square fit was performed simultaneously against all experimental data using the Mathematica function Find Minimum to minimize the following objective function:


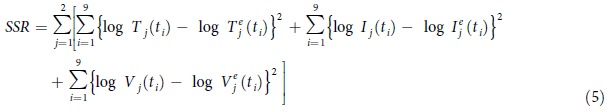


where *T*_*i*_(*t*_*i*_), *I*_*i*_(*t*_*i*_) and *Vi*(*t*_*i*_) are the model-predicted values for uninfected and infected cells and p24 viral load, given by the solution of Eqs. (2, 3, 4) at measurement time 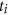 (*t*_*i*_ = 0,1,2,…,8d). Index *j* is a label for the two experiments without and with IFN. The variables with superscript “e” are the corresponding experimental measurements of those quantities. Experimental measurements below the detection limit were excluded when computing the *SSR*. The uncertainty of the cell- and virus-counting method used in our work is log-normally distributed, and for this reason we used the log of the cell concentration and viral load in fitting our model to the experimental data. This also allows to equally weight the difference in cell numbers on that in viral load in our objective function.

## Additional Information

**How to cite this article**: Ikeda, H. *et al.* Quantifying the Antiviral Effect of IFN on HIV-1 Replication in Cell Culture. *Sci. Rep.* **5**, 11761; doi: 10.1038/srep11761 (2015).

## Supplementary Material

Supplementary Information

## Figures and Tables

**Table 1:** Parameter estimated by mathematical-experimental analysis.

Parameter Name	Symbol	Unit	aHIV-1 NL-AD8	aHIV-1 NL-AD8 with IFN	aHIV-1 NL4-3	aHIV-1 NL4-3 with IFN
Parameters obtained from simultaneous fit to full *in vitro* dataset						
Rate constant for infection	*β*	(p24 ng/well·day)^−1^	(6.73 ± 2.00) × 10^–3^		(8.76 ± 3.74) × 10^–3^	
Death rate of infected cells	*δ*	day^−1^	1.56 ± 0.16		1.80 ± 0.19	
Production rate of total virus	*p*	10^–3^ × p24 ng/well·(cell·day^−1^)	2.55 ± 0.68		3.92 ± 1.19	
Antiviral effect of IFN on *de novo* infection	*η*	—	No inhibition	0.47 ± 0.06	No inhibition	0.33 ± 0.10
Antiviral effect of IFN on virus production	*ε*	—	No inhibition	0.06 ± 0.9	No inhibition	0.15 ± 0.11
Initial amount of virus inoculation	*V*(0)	p24 ng/well	0.79 ± 0.57	2.03 ± 1.07	2.24 ± 1.70	1.23 ± 0.29
Quantities derived from fitted values						
Half-life of infected cells	log2/*δ*	days	0.45 ± 0.05		0.39 ± 0.04	
Viral burst size	(1–*ε*)*p*/*δ*	10^–3^ × p24 ng/well·cell^−1^	1.69 ± 0.62	1.59 ± 0.65	2.14 ± 0.43	1.76 ± 0.59
Malthusian coefficient	*Θ*	day^−1^	4.97 ± 0.14	2.88 ± 0.09	7.24 ± 0.86	4.79 ± 0.52
Basic reproductive number	*R*_0_	—	13.1 ± 0.33	6.37 ± 0.43	19.0 ± 6.88	7.24 ± 0.86

^a^Values are averages and standard deviations for three independent experiments.
