# Corrigendum: Quantifying the Antiviral Effect of IFN on HIV-1 Replication in Cell Culture

**DOI:** 10.1038/srep15679

**Published:** 2015-10-22

**Authors:** Hiroki Ikeda, Ana Godinho-Santos, Sylvie Rato, Bénédicte Vanwalscappel, François Clavel, Kazuyuki Aihara, Shingo Iwami, Fabrizio Mammano

Scientific Reports 5: Article number: 1176110.1038/srep11761; published online: 06292015; updated: 10222015

In this Article, an additional affiliation for Ana Godinho-Santos was omitted. The additional affiliation is listed below:

Research Institute for Medicines (iMed.ULisboa), Faculty of Pharmacy, University of Lisbon, Lisbon, Portugal

