# Supplementary material for: Quantifying the Antiviral Effect of IFN on HIV-1 Replication in Cell Culture
Source: Sci Rep. 2015 Jun 29;5:11761. doi: 10.1038/srep11761 (PMC4483772; doi:10.1038/srep11761)
Supplement: Supplementary Information [file srep11761-s1.pdf]

Quantifying the Antiviral Effect of IFN on HIV-1 Replication in Cell Culture - supplementary information -

Hiroki Ikeda[1†], Ana Godinho-Santos[2†], Sylvie Rato[2], Bénédicte Vanwalscappel[2,3], François Clavel[2,3], Kazuyuki Aihara[4,5], Shingo Iwami[1,6,7*‡], Fabrizio Mammano[2,3‡]

[1] Department of Biology, Kyushu University, Fukuoka 812-8581, Japan. [2] INSERM, U941, Paris, France. [3] Univ Paris Diderot, Sorbonne Paris Cité, IUH, Paris, France. [4] Institute of Industrial Science, The University of Tokyo, Meguro-ku, Tokyo, Japan. [5] Graduate School of Information Science and Technology, The University of Tokyo, Bunkyo-ku, Tokyo, Japan. [6] PRESTO, JST, Kawaguchi, Saitama 3320012, Japan. [7] CREST, JST, Kawaguchi, Saitama 3320012, Japan.

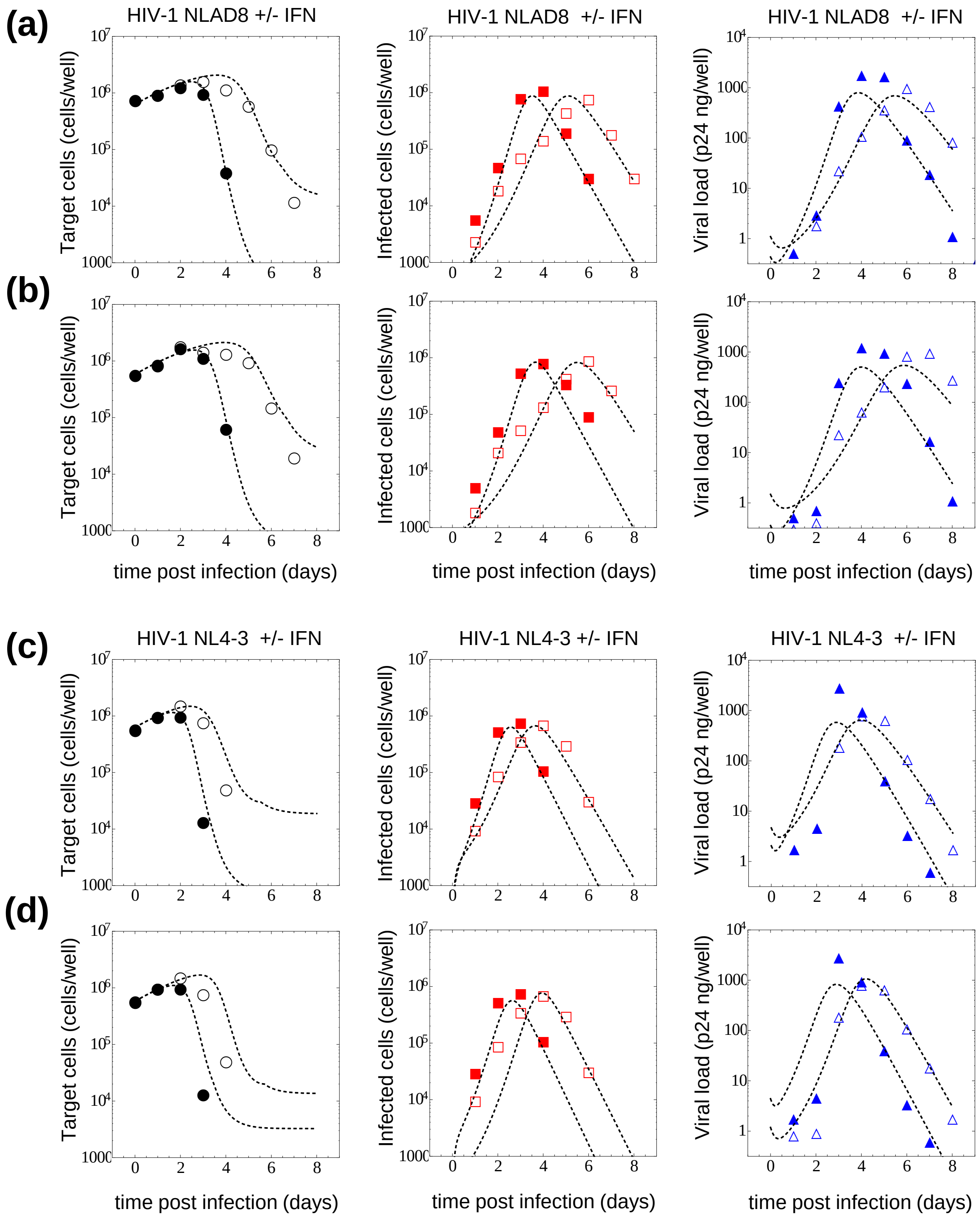

Supplemental Figure 1. HIV-1 replication in the absentce and presence of IFN.

MT4R5 cells were inoculated with 10 ng of p24 of HIV-1 NL-AD8 (**a** and **b** represent independent experiments) or HIV-1 NL4-3 (**c** and **d**), without or with IFN The number of intracellular Gag-negative and -positive MT4C5 cells per well and the amount of p24 viral protein (ng/well) in the culture supernatant were measured daily from $t = 0$ to 8. The bullet (●■▲) and open (○□△) symbols show the representative experimental data from one experiment in the absence and presence of IFN, respectively. The best fit of the mathematical model , Eqs.(2-4), to the data is depicted as broken and dotted lines in the absence and presence of IFN, respectively.

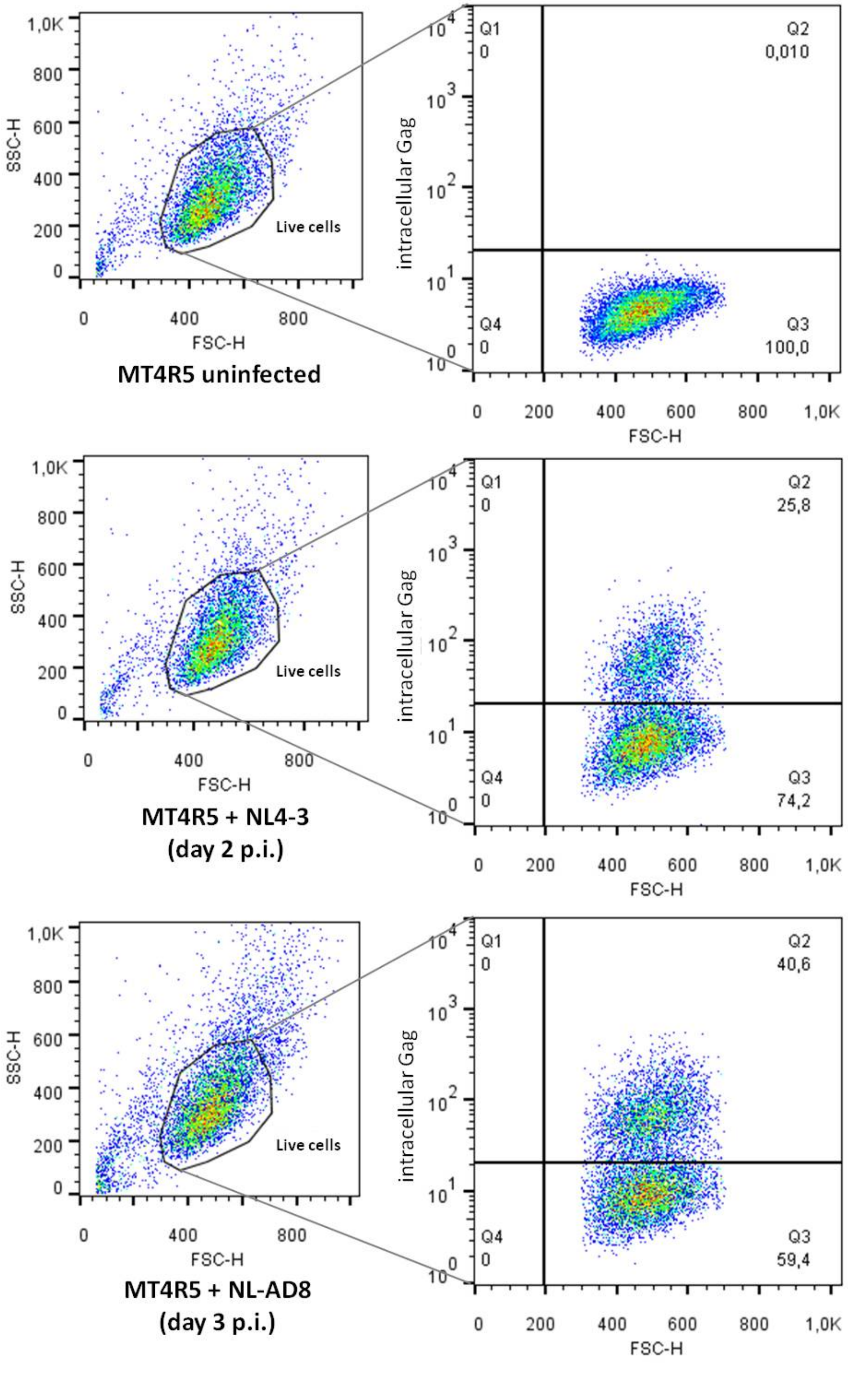

Supplemental Figure 2. FACS analysis of uninfected and HIV-infected cells.

To monitor virus spread in culture and quantify the percentage of infected and uninfected cells over time, cells were collected, permeabilized, and incubated in the presence of an anti-Gag antibody (as detailed in reference 25). Cells were then analyzed by FACS, by gating on live cells (left panels), and measuring the percentage of intracellular-Gag positive and negative cells (right panels). Data reported here are from one representative experiment, comparing the profile of uninfected MT4R5 cells, to cells infected by NL4-3 (at day 2 post-infection) or infected by NL-AD8 (at day 3 post-infection).
